# The Italian mental health-care reform: public health lessons

**DOI:** 10.2471/BLT.18.216002

**Published:** 2018-11-01

**Authors:** Corrado Barbui, Davide Papola, Benedetto Saraceno

**Affiliations:** a World Health Organization Collaborating Centre for Research and Training in Mental Health and Service Evaluation, Section of Psychiatry, University of Verona, Policlinico GB Rossi, Piazzale Scuro 10, 37134 University of Verona, Italy.; b Lisbon Institute of Global Mental Health, Lisbon, Portugal.

Psychiatric hospitals often represent the main and sometimes only treatment option available for people with mental disorders.^1^ This treatment model contradicts the balanced-care model, which suggests that a comprehensive mental health system needs to include and integrate outpatient community care, acute in-patient care in general hospitals and community-based residential care.^2^ In Italy, the transition from a hospital-based system of care to a model of community mental health care started in 1978 with a reform that led to the gradual closing of psychiatric hospitals. We now reflect on the public health lessons from this transition, which may be useful for countries aiming to implement a community mental health-care model.

The first lesson is that psychiatric hospitals are not essential. In Italy, the number of people in psychiatric hospitals declined from 78 538 individuals in 1978 to 7704 in 1998. By 2000, all psychiatric hospitals were closed.^3^ Currently, no beds in psychiatric hospitals are available; however, there are 10 beds in psychiatric wards located in general hospitals per 100 000 population and 46 beds in community residential facilities per 100 000 population, with wide differences between geographical areas.^4^

Second, decreasing the number of psychiatric beds does not necessarily lead to increased suicide rates. According to the Organisation for Economic Co-operation and Development (OECD) database, suicide rates have remained stable in Italy: in 1978 there were 7.1 suicides per 100 000 population, while in 2012 there were 6.3 suicides per 100 000 population.^5^

Third, decreasing the total number of psychiatric beds does not lead to increased compulsory admissions. After the implementation of the Italian reform, the absolute number of compulsory admissions progressively declined, from more than 20 000 in 1978 to less than 9000 in 2015.^6^ Similarly, the proportion of compulsory psychiatric admissions progressively declined between 1978 and 2005 and remained stable thereafter, accounting in 2015 for less than 5% (8815) of all 187205 psychiatric admissions.^6^

Fourth, decreasing the total number of psychiatric beds does not lead to increased use of psychiatric forensic facilities. According to the few available data, in 1980 there were 1424 psychiatric patients placed in forensic psychiatric hospitals; in 1987 there were 977 and in 2012 there were 1264.^7^ In 2016, after the phasing out of forensic psychiatric hospitals, there were 541 individuals placed in new residential facilities providing intensive mental health care to socially dangerous individuals with mental disorders. Additionally, there were 201 individuals with mental disorders placed in psychiatric units in prison, yielding an overall number of 742 people for the year 2016.^7^

Fifth, the risk of custody, rather than rehabilitation, is not confined to psychiatric hospitals. Italy has invested in psychiatric beds placed in community residential facilities. However, the average length of stay in these facilities is almost two years, with high regional variability.^8^ This result may suggest that these facilities, rather than focusing on rehabilitation, only provide long-stay residential services. This explanation was also suggested by a survey that showed that almost half the patients in Italian residential facilities were totally inactive, not even assisting with their facility’s daily activities.^9^

Sixth, consolidation of this transition process may be an uncertain phase. As Italian psychiatry progressed from the stage of change to a consolidation and maintenance phase, the motivation and morale of mental health staff has progressively changed. In the early stages of a service reform, small groups may champion the main proposals and recruit support from other professionals and stakeholder groups.^10^ During this phase, enthusiasm and motivation is high. However, after a series of ground-breaking initiatives, the Italian mental health system now needs to systematize these changes so that they can be consolidated. However, maintenance activities are inevitably considered less attractive, professionals are less likely to be rewarded for their commitment to the service and are seldom involved in new initiatives for further improvement. Italy currently is in this consolidation phase, with fewer financial resources, and declining motivation and morale among mental health professionals.

Countries aiming to implement a community mental health-care model may want to consider these public health lessons. Policy-makers and health-care providers should note that closing psychiatric hospitals was not the main objective of the Italian reform; rather, it was to ensure that citizens with mental disorders would be treated just as other patients. This principle has revolutionized the role and focus of psychiatry in Italy, from custody, coercion and segregation to treatment and care. All the changes to the Italian mental health system have been a consequence of this paradigm shift.
